# Rapid Detection of Getah Virus Antibodies in Horses Using a Recombinant E2 Protein-Based Immunochromatographic Strip

**DOI:** 10.3390/ani14162309

**Published:** 2024-08-08

**Authors:** Dengke Zhong, Jiayang Zheng, Zhiyong Ma, Yan Wang, Jianchao Wei

**Affiliations:** 1 Department of Animal Science and Technology, Shanghai Vocational College of Agriculture and Forestry, Shanghai 201699, China; 211506@shafc.edu.cn; 2 Shanghai Veterinary Research Institute, Chinese Academy of Agricultural Sciences, Shanghai 200241, China; asd126163@163.com (J.Z.); zhiyongma@shvri.ac.cn (Z.M.); 3 Technical Center for Animal, Plant and Food Inspection and Quarantine of Shanghai Customs, Shanghai 200135, China

**Keywords:** Getah virus, colloidal immunochromatographic strip, horse, rapid detection

## Abstract

**Simple Summary:**

Getah virus (GETV) is an important zoonotic pathogen with an extensive host range, including horses, pigs, cattle, sheep, birds, and humans, resulting in substantial losses in the livestock and agricultural industries; as such, the prevalence and impact of GETV are significant concerns in China. In this study, the antigen domain of the E2 glycoprotein of GETV was expressed and purified in the *Escherichia coli* expression system, and an on-site immunochromatographic strip (ICS) was successfully developed to detect GETV antibodies in horses. The ICS has the advantages of being rapid, sensitive, specific, and is an effective method for the detection of GETV in horses that does not rely on special equipment or skilled personnel and can be used for the field diagnosis of GETV in horses.

**Abstract:**

The prevalence and impact of Getah virus (GETV) are significant concerns in China. GETV can infect a wide range of animals, including horses, pigs, sheep, cattle, birds, and humans, resulting in substantial losses in the livestock and agricultural industries. GETV infection can cause the development of ulcers and inflammation in the mouth and gums of horses, which result in pain and discomfort and lead to symptoms such as reduced appetite, drooling, and difficulty chewing. As a result, there is a pressing need for efficient and rapid disease diagnosis methods. However, the currently available diagnostic methods have limitations in terms of operational time, equipment, and the experience of the individuals using them. In this study, a rapid, specific, and sensitive detection method was developed using a colloidal gold-based immunochromatographic strip (ICS) for the detection of antibodies against GETV in horses. To prepare the ICS, the antigen domain of the E2 glycoprotein of GETV was expressed using the *Escherichia coli* expression system after analysis with DNAstar v7.1 software. The nitrocellulose membrane was coated with rE2 protein or SPA to form the test line and control line, respectively. After optimizing the reaction conditions, the sensitivity, specificity, and repeatability of the strip were verified. The results showed that the test strip had a detection limit of up to 1:320 dilutions for GETV-positive serum, with no cross-reactivity observed with other equine-susceptible pathogens such as equine arteritis virus (EAV), equine herpesvirus-1 (EHV-I), equine infectious anemia virus (EIAV), equine influenza virus (EIV), African horse sickness virus (AHSV), and Japanese encephalitis virus (JEV). Furthermore, the ICS exhibited a concordance rate of 94.0% when testing 182 clinical serum samples compared to the virus neutralization test. Overall, this ICS diagnosis method will be an effective tool for the rapid detection of GETV in the field.

## 1. Introduction

Getah virus (GETV), belonging to the family *Togaviridae*, is a member of the genus *Alphavirus* and is an important zoonotic pathogen with an extensive host range that causes human and animal infections [1,2]. The genome of GETV is a single positive-stranded RNA of 11–12 kb length, which encodes nonstructural proteins (NSP1–4) and structural proteins C, E3, E2, 6K, and E1 [3,4]. The E2 protein is the main functional protein involved in infection and triggering host immune responses [2]. Recombinant E2 (rE2) protein also can be used as the antigen for an indirect enzyme-linked immunosorbent assay (ELISA) for horse GETV [5,6]. Additionally, rE2 protein has a stronger reaction with infected horse sera than rE1 protein in both ELISA and Western blotting [5].

GETV has a wide geographical distribution in various regions, ranging from Eurasia to Southeast and far-Eastern Asia, the Pacific islands, and Australasia [7,8,9,10]. It primarily circulates among animals through mosquito species such as *Culex* and *Aedes*, depending on the geographic region [10], with horses acting as one of the main reservoir hosts [11]. Other domestic and wild animals including cattle, pigs, and foxes can also be infected [12,13,14,15].

The clinical signs in horses infected with GETV often include rashes on the skin, pyrexia, and edema in the legs [16,17]. There are several GETV diagnosis methods, including virus neutralization (VN), hemagglutinin inhibition, and complement fixation tests [18]. At present, ELISA is currently the most commonly used method and there is no commercial antigen test, so the etiological detection of GETV mainly includes virus isolation and virus gene amplification [2]. However, traditional diagnosis methods, including serological assays and molecular methods such as polymerase chain reaction, always rely on laboratory testing to detect specific antibodies and viral genetic material in the blood or other bodily fluids.

GETV has resulted in six major outbreaks in India and Japan, causing huge economic losses [19,20]. In China, GETV has been found to be prevalent in many species, but was not detected in horses until 2018 [21]. With the rapid development of the racehorse industry, there are many more horses in China now. GETV is a potential threat to horses in China because domestic horses have not been vaccinated widely against GETV infection [7]. As a result, there is a pressing need for the development of efficient and rapid GETV diagnosis methods in order to obtain a better understanding of the prevalence of GETV in the horse population in China. 

## 2. Materials and Methods

### 2.1. Virus and Serum Samples

The GETV standard-positive serum, GETV standard-negative serum, and serum samples for specificity testing, including equine arteritis virus (EAV), equine herpesvirus-1 (EHV-I), equine infectious anemia virus (EIAV), equine influenza virus (EIV), African horse sickness virus (AHSV), and Japanese encephalitis virus (JEV), were prepared by the China Animal Health and Epidemiology Center (Shanghai Branch). The anti-His-tag monoclonal antibody was obtained from Shanghai Nuolong Biotechnology Co., Ltd. (Shanghai, China). Nitrocellulose (NC) membranes, conjugate pads, sample pads, absorbent paper, and polyvinyl chloride sheets were purchased from Shanghai Joey Biotechnology Co., Ltd. (Shanghai, China).

### 2.2. Construction of Escherichia coli (E. coli)-Expressing Recombinant GETV E2

The amino acid sequence of the E2 glycoprotein of GETV strain SH05-6 was obtained from GenBank (accession number: ABV68929.1). The E2 protein (422 Amino acids) was analyzed using the Protean program of DNAstar v7.1. The sequence encoding 292 amino acids, which is composed of three antigenic domains: domain A (A17-P133), domain B (P172-H232), and domain C (P268-L341), was optimized and synthesized according to the codon of *E. coli*, and subcloned into the expression vector pCold I. The resulting plasmid construct containing the GETV E2 gene was confirmed using DNA sequencing. The recombinant plasmid was then transformed into *E. coli* BL21(DE3)-competent cells, and the resulting transformants were inoculated onto Luria–Bertani (LB) agar plates supplemented with ampicillin (100 μg/mL). Each individual colony of the transformants was incubated and further inoculated into LB broth containing ampicillin overnight at 37 °C; the samples were cultured at 180 rpm until the optical density at 600 nm (OD600) reached 0.6–0.8. Recombinant protein expression was induced by the addition of isopropyl-β-D-thiogalactoside (IPTG) with a final concentration of 1 mM. The culture was then incubated for 6 h, as previously determined [3].

### 2.3. Purification of Protein

The induced bacterial culture was harvested using centrifugation at a speed of 12,000× *g* for 30 min to pellet the cells. The cell pellet was then resuspended in an appropriate lysis buffer (10 mmol/L imidazole, 50 mmol/L NaH_2_PO_4_, 300 mmol/L NaCl ph 8.0), supplemented with protease inhibitors, and lysed through freeze–thaw cycles. The lysate was clarified via centrifugation, and the recombinant GETV E2 protein was purified from the supernatant. The purification process involved the use of Ni-NTA resin, which selectively binds to the His-tag on the recombinant protein. The purified protein was confirmed by SDS-PAGE and Western blotting analysis. The recombinant GETV E2 protein was stored at −80 °C for long-term use.

### 2.4. Western Blotting

Western blotting was conducted following previously described methods [3]. The purified protein *rE2* was separated using a 12% SDS-PAGE gel and subsequently transferred onto an NC membrane. The membrane was blocked with 5% skim milk and then incubated with anti-His-*tag* monoclonal antibody (dilution: 1:10,000), followed by incubation with an HRP-conjugated goat anti-mouse IgG antibody (dilution: 1:5000) or *GETV-positive horse serum* (1:500). The signals were visualized using the Western blotting kit for a duration of 2 min.

### 2.5. Preparation of the Colloidal Gold-Labeled Suspensions

The pH of the gold solution was adjusted by adding varying amounts of 0.2 mol/L K_2_CO_3_ to change the pH. Moderate amounts of labeled Staphylococcal protein A (SPA) were added and the 400–700 nm wavelength range was measured. Then, the pH of 7 EP tubes containing 1 mL of gold solution was adjusted to the optimal labeling pH using 14 mL of 0.2 mol/L K_2_CO_3_ solution. Different amounts of SPA were added to each EP tube and the absorption peaks were measured to determine the optimal labeling amount for the SPA.

### 2.6. Preparation of the Immunochromatographic Strip

**Figure 1:**
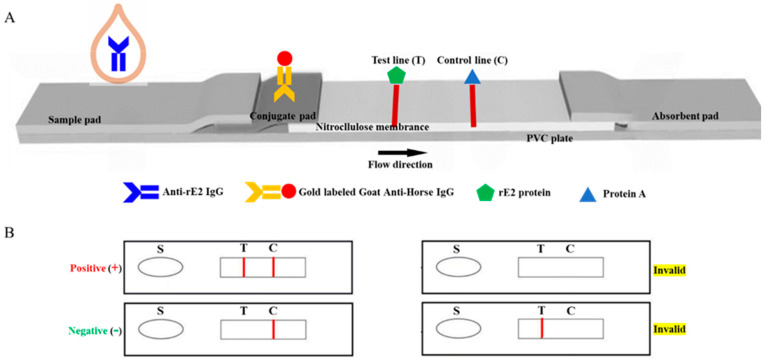
Schematic diagram of the immunochromatographic strip (ICS) test for detecting GETV antibodies in horse. (**A**) Structure of the ICS. (**B**) Interpretation of the results using ICS. A positive sample shows two red bands on the membrane strip; a negative sample shows only one band on the control line. If there is no colored band at all or there is only one colored band on the test line, the test is invalid. C, control line; T, test line; S, sampling hole.

The components of the ICS are illustrated in Figure 1. The strip consists of four compartments: a sample pad, a conjugate pad, an NC membrane, and an absorbent pad. On the NC membrane, rE2 (0.1 mg/mL) and SPA (1.0 mg/mL, Sigma, Louis, MO, USA) were applied to form the test line and control line, respectively. This process was performed using an XYZ3050 dispense workstation, followed by drying the NC membrane at 37 °C for 1 h before storing it at 4 °C. The test line and control line were positioned 0.5 cm apart at the center of the membrane. The conjugate pad, made of a glass fiber membrane, was treated with goat anti-horse IgG–colloidal gold conjugate. After treatment, it was dried under vacuum. All of the components of the ICS kit, arranged in the proper order as depicted in Figure 1, were affixed to a backing plate (300 mm × 25 mm, SM31-25, Shanghai Kinbio Biotechnology Co., Ltd., Shanghai, China). Subsequently, the plate was sliced into 4 mm-wide strips using an automatic cutter. Each strip was then assembled onto a plastic cassette. The assembled strips were stored within a broad temperature range of 4–30 °C prior to use.

### 2.7. Specificity and Sensitivity Evaluations

The GETV standard-positive serum was diluted in a series of ratios of 1:10, 1:20, 1:40, 1:80, 1:160, 1:320, 1:680, and 1:1280 with 0.01 mol/L PBS. The same volume of standard-positive serum and standard-negative serum with different dilutions was added onto the test pad to evaluate the sensitivity of the test strip. Then, 100 µL of each prepared serum sample was added dropwise onto the sample pad, and pictures were taken after 10 min of incubation at room temperature. 

In addition, various other equine-susceptible pathogens, namely, EHV-I, EAV, EIV, EIAV, AHSV, and JEV, were introduced to test the specificity of the strips using the same procedure described above. 

### 2.8. Clinical Application of the ICS

A total of 182 clinical horse serum samples were collected and tested for GETV using the ICS test and VN test. In brief, the inactivated test serum was prepared at concentrations from 1:2 to 1:32. Then, the test samples were mixed with the virus and incubated for 1 h at 37 °C. The obtained mixture was then added to BHK-21 cells and incubated at 37 °C under 5% CO_2_ for 72 h. The results of the two methods were compared and the coincidence rate was calculated as follows: [(true positive + true negative)/(true positive + true negative + false positive + false negative) × 100%].

## 3. Results

### 3.1. Preparation and Identification of rE2

**Figure 2:**
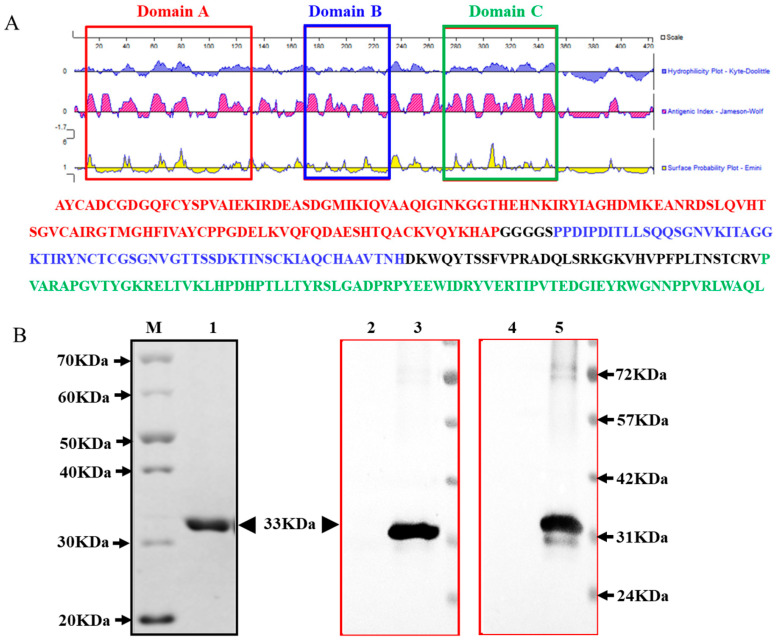
Design, preparation, and identification of recombinant protein rE2. (**A**) Amino acid sequence of the E2 protein of Getah virus strain SH05-6 was obtained from GenBank (accession number: EU015066.1) and included three functional domains. Antigenic domains of Getah virus E2 glycoprotein were analyzed using the Protean program of DNAstar v7.1. Colored boxes indicate domains A, B, and C, which were predicted in reference to those of Chikungunya virus [22]. (**B**) Production and identification of recombinant protein rE2.M: protein marker. Lane 1: Sodium dodecyl sulfate–polyacrylamide gel electrophoresis analysis of purified rE2 (33 kDa) expressed by vector pCold I. Lanes 2 and 4: Western blotting analysis of the *E. coli*-containing pCold using anti-His monoclonal antibody (Lane 2) and GETV-positive horse serum (Lane 4). Lanes 3 and 5: Western blotting analysis of the *E. coli*-containing pCold-E2 using anti-His monoclonal antibody (Lane 3) and GETV-positive horse serum (Lane 5).

GETV E2 glycoprotein was highly conserved and composed of three domains (A, B, and C).The three antigenic domains were considered useful antigens for serological diagnoses [3,5,6,12]. In order to efficiently express recombinant protein rE2, the E2 sequence was optimized by removing the hydrophobic domain and intracellular structure, followed by codon optimization. The optimized sequence was successfully cloned into the pCold I vector and expressed through the *E. coli* system. Then, the Ni-NTA resin was employed for purification. The SDS-PAGE (Figure 2B: Line 1) and Western blotting analyses (Figure 2B: Lines 2, 3, 4, and 5) demonstrated that the expressed GETV E2 protein was successfully expressed and purified with a molecular weight of 33 kDa, exhibiting a strong reaction with the anti-His monoclonal antibody (Figure 2B: Line 3) and GETV-positive horse serum (Figure 2B: Line 5).

### 3.2. Preparation of the Colloidal Gold-Labeled Suspensions

It was found that the highest peak absorption value was achieved when 14 mL of 0.2 mol/L K_2_CO_3_ was added, corresponding to a pH of 10.0. This indicates that the optimal labeling pH for the colloidal gold solution is pH 10. The results showed that the EP tube with the highest absorption peak corresponded to the addition of 18 μg of SPA (the optimal labeling amount of the protein dose corresponding to the highest absorption peak is 110%). Therefore, the optimal labeling amount for SPA is 20 μg/mL. The colloidal gold solution was adjusted to the previously determined optimal labeling pH of 10.0 using 0.2 mol/L K_2_CO_3_. The optimal labeling amount of 20 μg/mL of SPA was added. After binding, the OD400–700 nm range was measured. The spectral absorption curve was found to shift to the right, indicating the successful labeling of the gold-conjugated SPA antibody to the colloidal gold particles.

### 3.3. Sensitivity Evaluation of ICS

**Figure 3:**
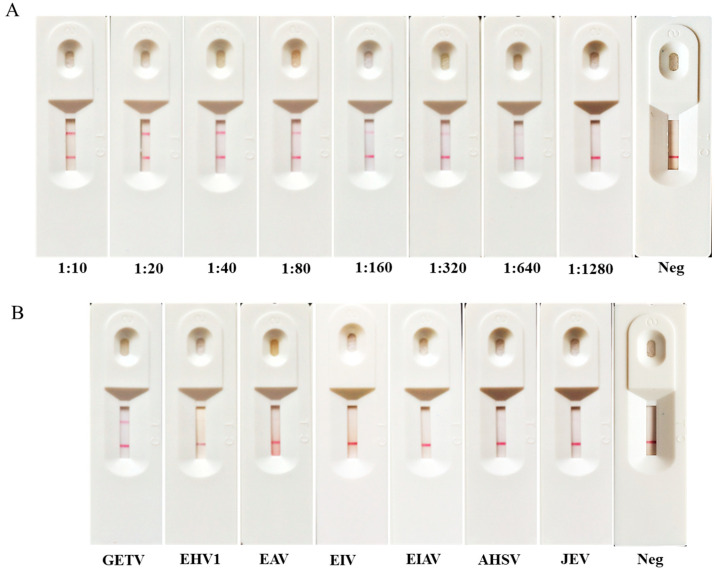
Sensitivity and specificity testing of the ICS assay. (**A**) Sensitivity of the ICS. GETV-positive serum was diluted from 1:10 to 1:1280 to determine the sensitivity of the ICS. The detection limit of the test strip was up to 1:320 dilutions for the GETV-positive serum. Neg: negative horse serum. (**B**) Specificity of the ICS. Sera positive for different horse viruses (EHV1, EAV, EIV, EIAV, AHSV, and JEV) were used to evaluate the specificity of the ICS. The ICS did not cross-react with other viral-positive sera.

To investigate the sensitivity of the ICS, we made a series of dilutions of GETV standard-positive serum in ratios of 1:10, 1:20, 1:40, 1:80, 1:160, 1:320, 1:680, and 1:1280 with 0.01 mol/L PBS (Figure 3A). The negative horse serum was diluted in the same way as the control. No red line appeared at the T position for the negative horse serum sample, which confirmed that the negative horse serum had no GETV antibodies. On the other hand, a clearly visible solid red line was observed until the 1:320 dilution, indicating that the test strip had a detection limit of up to 1:320 dilutions for the GETV-positive serum samples.

### 3.4. Specificity Evaluation of ICS

The horse positive serum of other horse viruses were used to test the specificity of the ICS. The results indicated that horse sera containing GETV-positive antibodies showed positive results, while the other anti-sera that were positive for other viruses showed negative results (Figure 3B), demonstrating that the ICS exhibits high specificity for GETV and no cross-reactivity with other pathogenic horse viruses.

### 3.5. Comparison with Neutralization Test

A total of 182 horse serum samples were collected from seven horse farms in Shanghai from 2018 to 2020. These samples were subjected to testing using both our developed ICS and VN methods. Out of the 182 samples tested using ICSs, 59 (32.4%) were found to be positive for GETV antibodies and 123 (67.6%) were negative. The same samples were tested using VN, and the findings showed that 52 (28.6%) of the samples were positive for GETV and 130 (71.4%) were negative. The coincidence rate of detection between these two methods was determined to be 94.0% (Table 1). This high coincidence rate of the ICS and VN test results indicates that the ICS can be as effective and reliable for detecting GETV antibodies as the current standard VN test.

## 4. Discussion

GETV is a zoonotic virus that can infect both humans and animals [10]. It can cause abortions in pigs and fever, skin rash, and lymph node enlargement in horses. Multiple reports have documented the occurrence of GETV infections in racehorses and pig populations in countries such as Japan, India, and China [12,20,23,24], indicating the prevalence of GETV in these countries. With the improvement in people’s living standards, horse-related sports and leisure industries have flourished in China, leading to an increasing horse population. Therefore, strengthening GETV monitoring, particularly for emerging diseases, is crucial for maintaining the health and sustainable development of related industries. So far, no highly effective preventive or treatment measures have been developed for diseases caused by GETV. Therefore, it is crucial to take effective prevention and control measures against GETV, such as mosquito control and vaccine development. Early diagnosis and treatment are also vital for individuals with GETV infections.

There are several diagnostic methods currently available for the detection of zoonotic GETV infections in both humans and animals, such as reverse transcription polymerase chain reaction [24,25], ELISA, and indirect fluorescent antibody assays [26]. It is important to note that these diagnostic methods may be restricted by factors such as a lack of well-trained individuals, laboratory capabilities, and specific equipment [27,28]. There has been a growing interest in the development of rapid detection techniques for GETV that aim to provide timely and accurate results, enabling early detection and the prompt implementation of control measures; one such method is the colloidal gold rapid detection method. In this study, the development of accurate and specific rapid detection methods for GETV is essential for the effective monitoring and prevention of GETV-related diseases. It is a user-friendly and cost-effective method that can provide rapid results, making it suitable for the large-scale screening of horse populations. The ICS method has been proven effective in the diagnosis of various infectious agents and has wide applications in the field [29,30]. It is developed using the rE2 protein of GETV, which is combined with colloidal gold to form an immunogold complex. Thus, the ICS method is a faster and more sensitive method for diagnosing and monitoring GETV antibodies in horse serum. The E2 glycoprotein contains an antigenic determinant and is considered a useful antigen for serological diagnoses [5]. Meanwhile, the rE2 protein conjugated with colloidal gold can specifically bind to GETV antibodies present in horse serum. These binding antibodies are then captured by immobilized SPA, forming a visible red band and thereby indicating the presence of GETV-positive antibodies in the samples.

Due to the lack of commercialized diagnostic kits for the serological diagnosis of GETV, the VN test was considered as the gold criteria for detecting GETV antibodies [18]. There are few publications regarding the detection of GETV antibodies in horses using ICS so far [5,6,26]. In this study, our method demonstrated high specificity and sensitivity, while the VN co-verification test results showed that the overall coincidence rate between ICS and VN was 94.0%, with 84.7% sensitivity and 98.4% specificity.

With the rapid development of the racehorse industry, GETV is a potential threat to horses in China. The seroprevalence of 32.4% was found for horses in Shanghai, was higher than 17% in India [20], but less than 54.3% in Xijiang [26] and 55.8–61.2% in Japan [19]. This innovative ICS will prove invaluable for the rapid and cost-effective diagnosis of GETV, eliminating the need for expensive laboratory equipment, which will benefit the prevention of GETV outbreaks in horses.

## 5. Conclusions

In this study, an on-site ICS for the rapid detection of GETV was developed and validated. It is an effective method for the detection of GETV in horses without relying on special equipment or skilled personnel, which will be used for the field diagnosis of GETV in horses in the near future.

## Figures and Tables

**Table 1:** Comparison of the results between immunochromatographic strip (ICS) and virus neutralization test (VN) for detection of GETV antibodies from horses in Shanghai, China.

	ICS	Positive	Negative	Total	Coincidence Ratio
VN					
Positive		50	9	59	84.7%
Negative		2	121	123	98.4%
Total		52	130	182	94.0%

Relative sensitivity = 50/59 = 84.7%, Relative specificity = 121/123 = 98.4%, Overall coincidence rate = (50 + 121)/182 = 94.0%.

## Data Availability

Data are contained within the article.
